# Tick-Borne Infection as a Precipitant of Guillain-Barré Syndrome: A Case of Lyme Neuroborreliosis

**DOI:** 10.7759/cureus.106652

**Published:** 2026-04-08

**Authors:** Justin Baik, Mohamed Said, Nicholas Helmstetter

**Affiliations:** 1 Internal Medicine, Western Michigan University Homer Stryker M.D. School of Medicine, Kalamazoo, USA; 2 Pediatric and Adolescent Medicine, Western Michigan University Homer Stryker M.D. School of Medicine, Kalamazoo, USA

**Keywords:** ascending paralysis, guillain-barre syndrome, guillain-barré syndrome, lyme neuroborreliosis, polyradiculoneuropathy, tick-borne infections

## Abstract

Overlapping clinical features between Lyme disease and Guillain-Barré Syndrome (GBS) can complicate diagnosis, and a definitive causal relationship has not been established. A 58-year-old woman experiencing unsheltered homelessness was referred to the emergency department by her street medicine physician with progressive symmetric weakness, unilateral facial nerve palsy, dysphagia, and dyspnea following tick bites obtained at her encampment in the woods. Workup showed elevated cerebrospinal fluid (CSF) protein with lymphocytic pleocytosis and positive serum Lyme serology tests, consistent with acute infection with Borrelia burgdorferi. Electromyography (EMG) demonstrated proximal demyelination, raising concern for concurrent GBS. She was treated with intravenous ceftriaxone and intravenous immunoglobulin (IVIG), resulting in gradual neurological improvement. This case underscores that Lyme neuroborreliosis can mimic or precipitate GBS-like neuropathy, and when overlap cannot be excluded, combined antibiotic and immunotherapy may be necessary. Early recognition is crucial to prevent respiratory or cardiac complications from overlapping Lyme and GBS pathology. This case underscores the importance of diagnosing and distinguishing GBS from Lyme neuroborreliosis when features overlap. Recognition of atypical findings, particularly inflammatory cerebrospinal fluid profiles, is essential to guide appropriate combined therapy and optimize neurological outcomes.

## Introduction

Lyme disease, caused by the spirochete Borrelia burgdorferi, is the most common vector-borne infection in the US and can disseminate to the nervous system, producing Lyme neuroborreliosis [1]. Neurologic manifestations may include cranial neuritis, radiculoneuritis, and polyradiculopathy. While Guillain-Barré syndrome (GBS) is an acute immune-mediated neuropathy most often triggered by infections such as Campylobacter jejuni, cytomegalovirus, or Epstein-Barr virus [2], Lyme disease is not yet established as a possible precipitant.

We report a 58-year-old woman with recently diagnosed Lyme disease presenting with symmetric ascending weakness, areflexia, dysphagia, and facial nerve palsy, consistent with GBS in the setting of Lyme neuroborreliosis. Her presentation illustrates the diagnostic challenge when Lyme infection mimics or potentially triggers immune-mediated demyelinating neuropathy.

## Case presentation

A 58-year-old woman experiencing unsheltered homelessness in a wooded area with a history of hypertension, type 2 diabetes, and juvenile inflammatory arthritis was referred to the emergency department by her Street Medicine registered medical practitioner for progressive weakness, facial droop, dysphagia, and shortness of breath following multiple tick bites for the past month.

Two days prior to admission, she presented to the emergency department (ED) after a fall from her bike and a persistent facial droop. A CT brain and spine was unremarkable, and she was discharged. Later in the day, her Street Medicine physician obtained laboratory studies, empirically initiated doxycycline due to high suspicion for Lyme disease, and referred her back to the ED when the Lyme serologies returned positive.

On admission, she reported over one month of worsening generalized weakness with recurrent falls, beginning in the lower extremities and ascending to involve the upper extremities. She became unable to ambulate or rise from a seated position and developed right-sided facial weakness involving the forehead. She endorsed mild distal paresthesia without bowel or bladder dysfunction, dysphagia, a weakened cough, headache, chills, and nausea, but denied fever or speech difficulty.

Vital signs were within normal limits. Neurologic examination revealed a right-sided complete facial nerve palsy. Upper extremity strength and tone were preserved, while lower extremity strength was reduced to 3+/5 bilaterally with absent patellar reflexes. Sensory examination demonstrated mildly diminished light-touch sensation in the distal right foot, with preserved pain and vibration sensation. Proprioception was reduced in the great toes bilaterally. Coordination was without prominent dysmetria, dyssynergia, dysdiadochokinesia, or ataxia, and she had a positive finger-to-nose test and a slow heel-to-shin.

Laboratory studies showed a normal white blood cell (WBC) count and an elevated erythrocyte sedimentation rate (ESR), whereas the EKG demonstrated normal sinus rhythm without heart block, and the chest X-ray was unremarkable. Her Lyme central nervous system (CNS) infection IgG antibody index and her serum IgG and IgM Lyme antibodies were elevated. The vaginal swab for gonorrhea, chlamydia, and trichomonas returned negative. Screening for HIV, Hepatitis B, and Hepatitis C also returned negative (Table 1).

**Table 1:** Laboratory studies CMP: Comprehensive Metabolic Panel; CO₂: Carbon Dioxide; BUN: Blood Urea Nitrogen; eGFR: Estimated Glomerular Filtration Rate; ALP: Alkaline  Phosphatase; AST: Aspartate Aminotransferase; ALT: Alanine Aminotransferase; CRP: C-Reactive Protein; CBC: Complete Blood Count; WBC : White Blood Cell (count); RBC: Red Blood Cell (count); MCV: Mean Corpuscular Volume; MCH: Mean Corpuscular Hemoglobin; MCHC: Mean Corpuscular Hemoglobin Concentration; RDW: Red Cell Distribution Width; MPV: Mean Platelet Volume; CSF: Cerebrospinal Fluid; RBC (in CSF): Red Blood Cells; PMNs %: Polymorphonuclear Leukocytes Percentage; IgM: Immunoglobulin M; IgG: Immunoglobulin G; HIV: Human Immunodeficiency Virus.

CMP	Patient value	Normal range
Glucose (mg/dL)	194	70-99
Sodium (mmol/L)	139	135-145
Potassium (mmol/L)	4.3	3.5-5.0
Chloride (mmol/L)	102	96-106
CO₂ (mmol/L)	24	22-29
Anion Gap (mmol/L)	13	8-16
Creatinine (mg/dL)	0.62	0.6-1.3
BUN	15	7-20
BUN/Creatinine Ratio	24	10-20
eGFR (mL/min/1.73m²)	>90	≥60
Calcium (mg/dL)	9.0	8.5-10.5
Phosphorus (mg/dL)	4.3	2.5-4.5
Magnesium (mg/dL)	2.1	1.7-2.4
ALP (U/L)	77	44-147
Albumin (g/dL)	3.7	3.5-5.0
Albumin/Globulin Ratio	1.3	1.1-2.2
AST (U/L)	18	10-40
ALT (U/L)	10	7-56
Total Bilirubin (mg/dL)	0.6	0.1-1.2
Total Protein (g/dL)	6.6	6.0-8.3
Globulin (g/dL)	2.9	2.0-3.5
CRP (mg/L)	25.2	<5
CBC	Patient value	Normal range
WBC (x10³/µL)	7.2	4.0-10.5
RBC (x10⁶/µL)	4.05	4.2-5.4
Hemoglobin (g/dL)	11.8	12-16
Hematocrit (%)	36.0	36-46
MCV (fL)	88.9	80-100
MCH (pg)	29.1	27-33
MCHC (g/dL)	32.8	32-36
RDW (%)	14.3	11-15
Platelets (x10³/µL)	266	150-450
MPV (fL)	10.1	7.5-11.5
CSF studies	Patient value	Normal range
CSF Glucose	129	40-70
CSF Protein	218	15-45
CSF Color	Colorless	Colorless
CSF Clarity	Clear	Clear
CSF Volume	3.5	0.5-4
CSF Nucleated Cells	56	0-5
CSF RBC	<2000	0
CSF PMNs %	1	0-6
Infectious disease	Patient value	Normal result/range
West Nile Virus IgM/IgG, CSF	Negative	Negative
Syphilis IgG	Negative	Negative
Lyme CNS IgG Antibody Index	Positive	Negative
Lyme CNS IgG Antibody Index Value	20.0	<0.9
Lyme CNS Infection IgG, CSF	Reactive	Non-reactive
Lyme Antibody (Serum)	>8.0	<0.9
Trichomonas	Negative	Negative
HIV-1/HIV-2 Antibody	Negative	Negative

**Figure 1:**
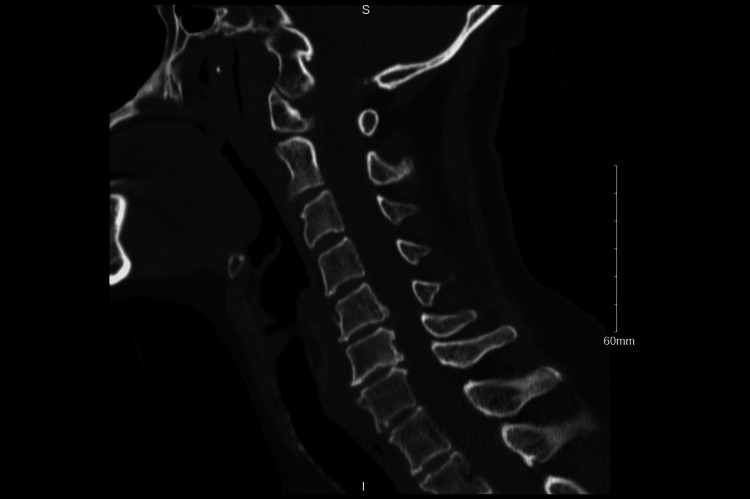
CT spine cervical without contrast No acute cervical spine fracture or traumatic malalignment.

**Figure 2:**
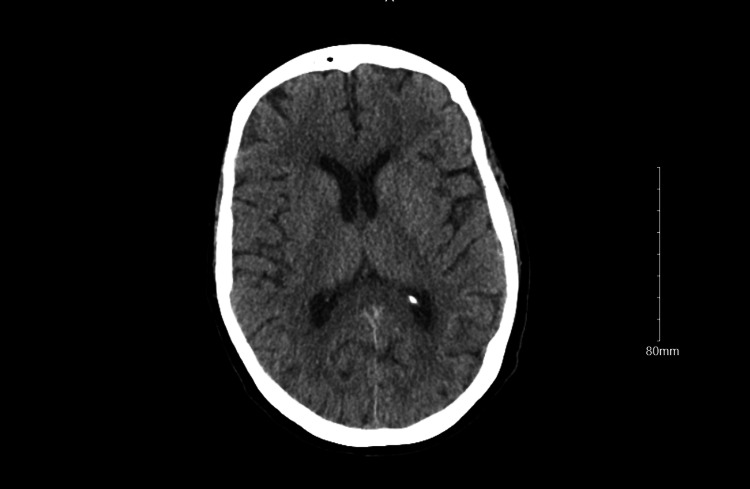
CT brain without contrast No acute intracranial abnormality.

CT of the head and cervical spine was unremarkable (Figures 1, 2).

**Figure 3:**
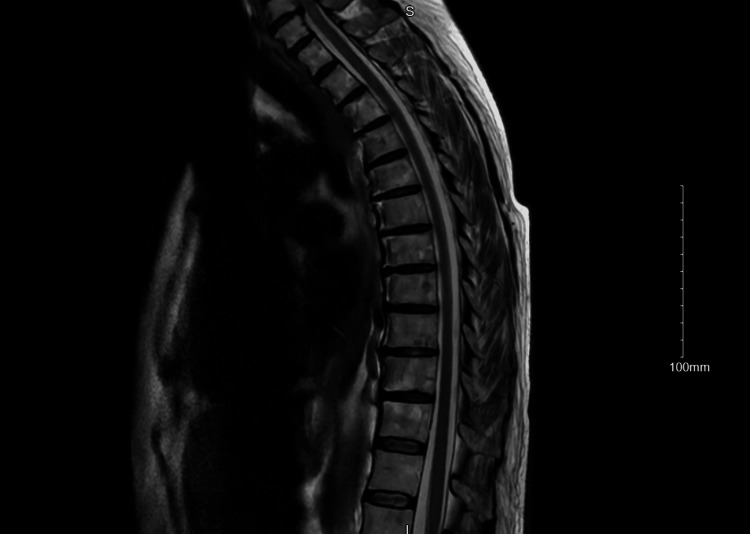
MRI spine complete without and with contrast No discrete lesion in the spinal cord. Multilevel degenerative changes.

MRI spine showed multilevel degenerative changes without any discrete lesions, moderate spinal canal stenosis at C6-7, and moderate neural foraminal stenosis bilateral at C5-6, C6-7, and L5-S1 (Figure 3). 

CSF analysis revealed markedly elevated proteins of 218 mg/dL (reference range: 15-45 mg/dL), elevated glucose of 129 mg/dL (reference range: 50-75 mg/dL), and elevated WBCs of 56 cells/µL (reference range: 0-5 cells/µL) with marked lymphocytic pleocytosis (96%). CSF cultures and meningoencephalitis polymerase chain reaction (PCR) testing were negative.

**Figure 4:**
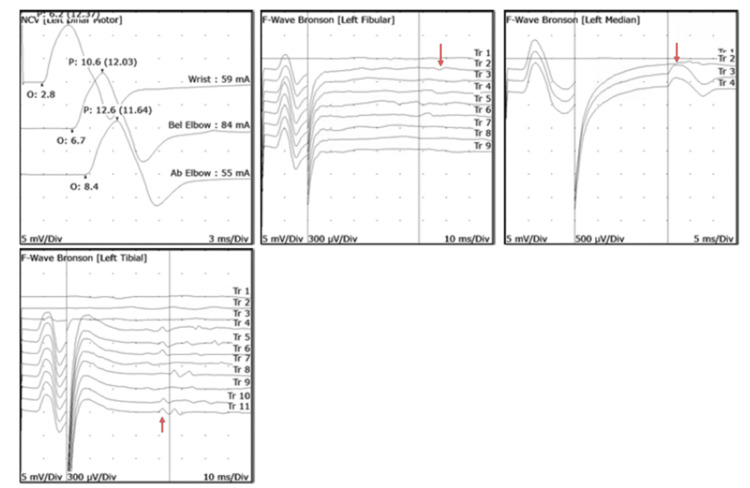
Electromyogram/Nerve Conduction Velocity (EMG/NCV) This is an abnormal study. The left median, tibial, and fibular f-wave responses are prolonged (red arrows). Motor and sensory responses show normal amplitudes and distal latencies/conduction velocities. This exam is consistent with an early proximal demyelinating polyneuropathy.

Given the symmetric ascending weakness, areflexia, and facial nerve involvement, GBS was initially considered. However, the marked lymphocytic pleocytosis was atypical for GBS and more consistent with Lyme neuroborreliosis, particularly early disseminated disease with radiculoneuritis and cranial neuritis. Electromyography demonstrated prolonged F-waves suggestive of proximal demyelination, raising concern for overlapping early GBS (Figure 4).

The patient was transitioned from doxycycline to intravenous ceftriaxone 2 g daily for 28 days. Given the concern for concurrent immune-mediated neuropathy, she also received a five-day course of intravenous immunoglobulin. Serial respiratory measurements remained stable. She demonstrated gradual neurologic improvement and was discharged to a subacute rehabilitation facility. At one-month follow-up, her neurologic examination had largely normalized.

## Discussion

We describe a case of a patient presenting with symmetric ascending weakness and facial palsy, ultimately found to have overlapping features of GBS and Lyme neuroborreliosis. While the patient met classic clinical and electromyography criteria for GBS, CSF analysis revealed marked lymphocytic pleocytosis, an atypical finding suggestive of coexisting inflammatory processes.

Lyme neuroborreliosis can manifest as polyradiculitis and cranial neuritis, with early disseminated infection affecting nerve roots. Key clinical features include symmetric limb weakness with preserved sensation, facial nerve involvement, and elevated CSF protein lymphocytes. The absence of significant sensory loss or autonomic dysfunction further supported Lyme radiculoneuritis in this patient. Although Borrelia burgdorferi infection may act as a trigger for immune-mediated neuropathies like GBS, definitive causal evidence is lacking, unlike established triggers such as Campylobacter jejuni, cytomegalovirus, and Epstein-Barr virus [2-4].

Proposed mechanisms for Lyme-associated neuropathy include direct inflammatory injury and molecular mimicry. Borrelia burgdoferi expresses outer membrane proteins that induce local inflammation, perineuritis, and demyelination of nerve roots [5]. Cross-reactive antibodies against bacterial antigens, including OspA and flagellar proteins, may target neuronal structures, contributing to chronic neurological or rheumatological symptoms [6,7]. Elevated CSF glucose could be explained by the high blood glucose at the time of her CSF analysis [8].

Management requires recognition of overlapping pathology. Lyme neuroborreliosis is treated with targeted antibiotics such as ceftriaxone [9], whereas GBS is managed with intravenous immunoglobulin or plasma exchange [10].

## Conclusions

Early identification is critical to begin appropriate management and optimize recovery. Patients are also at risk for cardiac conduction abnormalities from Lyme disease and respiratory compromise from GBS, warranting a low threshold for critical care consultation.

This case highlights the importance of considering tick-borne infections in patients with atypical GBS presentations, particularly in populations at a higher risk for exposure. Awareness of this overlap can guide timely diagnosis, appropriate treatment, and improved patient outcomes.
